# Both mTORC1 and mTORC2 are involved in the regulation of cell adhesion

**DOI:** 10.18632/oncotarget.3044

**Published:** 2015-01-23

**Authors:** Long Chen, Baoshan Xu, Lei Liu, Chunxiao Liu, Yan Luo, Xin Chen, Mansoureh Barzegar, Jun Chung, Shile Huang

**Affiliations:** ^1^ Jiangsu Key Laboratory for Microbes and Functional Genomics, Jiangsu Key Laboratory for Molecular and Medical Biotechnology, College of Life Sciences, Nanjing Normal University, Nanjing 210023, P. R. China; ^2^ Department of Biochemistry and Molecular Biology, Louisiana State University Health Sciences Center, Shreveport, LA 71130, USA; ^3^ Feist-Weiller Cancer Center, Louisiana State University Health Sciences Center, Shreveport, LA 71130, USA

**Keywords:** rapamycin, mTOR, cell adhesion, Akt, 4E-BP1

## Abstract

mTOR is a central controller for cell growth/proliferation and survival. Recent studies have shown that mTOR also regulates cell adhesion, yet the underlying mechanism is not known. Here we found that inhibition of mTOR by rapamycin reduced the basal or type I insulin-like growth factor (IGF-1)-stimulated adhesion of cancer cells. Further research revealed that both mTORC1 and mTORC2 were involved in the regulation of cell adhesion, as silencing expression of raptor or rictor inhibited cell adhesion. Also, PP242, an mTORC1/2 kinase inhibitor, inhibited cell adhesion more potently than rapamycin (mTORC1 inhibitor). Of interest, ectopic expression of constitutively active and rapamycin-resistant mutant of p70 kinase 1 (S6K1) or downregulation of eukaryotic initiation factor 4E (eIF4E)-binding protein 1 (4E-BP1) conferred resistance to rapamycin inhibition of cell adhesion, whereas expression of constitutively hypophosphorylated 4E-BP1 (4EBP1-5A) or downregulation of S6K1 suppressed cell adhesion. In contrast, neither genetic manipulation of Akt activity nor pharmacological inhibition of Akt affected cell adhesion. The results suggest that both mTORC1 and mTORC2 are involved in the regulation of cell adhesion; and mTORC1 regulates cell adhesion through S6K1 and 4E-BP1 pathways, but mTORC2 regulates cell adhesion via Akt-independent mechanism.

## INTRODUCTION

Cancer metastasis is recognized as the primary cause of death in most cancer patients. The metastatic process consists of a series of sequential and interrelated steps, including cancer cell detachment from the primary tumor, invasion of the extracellular matrix (ECM), intravasation of the vascular or lymph vessels, extravasation from the vessels of delivery, attachment to and proliferation within specific distant organs in the body [1, 2]. Inhibition of any of these steps may impair cancer metastasis formation. As attachment is a critical step during this process, intervening cancer cell adhesion should be an effective strategy to prevent cancer metastasis.

The serine/threonine (Ser/Thr) kinase mammalian target of rapamycin (mTOR) lies downstream of type I insulin-like growth factor (IGF-1) receptor and phosphatidylinositol 3′-kinase (PI3K), and is a central controller of cell growth/proliferation and survival [3, 4]. In mammalian cells, mTOR functions at least as two complexes (mTORC1 and mTORC2) [3, 4]. mTORC1 is composed of mTOR, mLST8 (also termed G-protein β-subunit-like protein, GβL, a yeast homolog of LST8), raptor (regulatory-associated protein of mTOR), and PRAS40 (proline-rich Akt substrate 40 kDa), and is rapamycin-sensitive [5–10]. mTORC2 consists of mTOR, mLST8, mSin1 (mammalian stress-activated protein kinase-interacting protein 1), rictor (rapamycin insensitive companion of mTOR), and protor (protein observed with rictor), and is rapamycin-insensitive, although long-term rapamycin treatment can inhibit mTORC2 in some cell types [5, 11–18]. Both mTORC1 and mTORC2 can be inhibited by ATP-competitive mTOR kinase inhibitors such as AZD8055 and PP242 [4]. In addition to their different sensitivity to rapamycin, mTORC1 and mTORC2 are activated by different stimuli and have distinct substrate specificity and functions. mTORC1 responds to growth factors, amino acids, energy and oxidative stress, but mTORC2 appears to be mediated only by growth factors [3, 4, 19]. mTORC1 phosphorylates p70 S6 kinase 1 (S6K1) and eukaryotic initiation factor 4E (eIF4E) binding protein 1 (4E-BP1), and controls protein and lipid synthesis, cell growth, proliferation, survival and motility [3–10, 20]. mTORC2 regulates phosphorylation or activity of Akt, glucocorticoid-inducible kinase 1 (SGK1), PKCα, focal adhesion proteins and small GTPases, and controls cell survival and actin cytoskeleton [11–18, 21–25]. Both mTORC1 and mTORC2 interact with a negative regulator DEPTOR [26].

S6K1 is defined to be primarily responsible for translation of mRNA species containing 5′ terminal oligopyrimidine (TOP) tracts [27, 28]. However, further studies have demonstrated that S6K1 turns on and off the eukaryotic initiation factor 3 (eIF3) translation initiation complex in a growth factor- and rapamycin-sensitive manner [29]. Activated S6K1 phosphorylates its translational targets, including the 40S ribosomal protein S6 and eIF4B, promoting translation initiation [29]. 4E-BP1 primarily functions as a suppressor of eIF4E [30]. Hypophosphorylated 4E-BP1 tightly binds to the cap-binding protein eIF4E and represses cap-dependent mRNA translation by blocking the interaction of eIF4E with the eIF4G protein and formation of the eIF4F initiation complex [30, 31]. The phosphorylation of 4E-BP1 at multiple site (Thr37, Thr46, Ser65, Thr70, Ser83 and Ser112) releases eIF4E to restore cap-dependent translation [30]. Both S6K1 and 4E-BP1/eIF4E pathways contribute to translation [29–31].

While extensive data have highlighted the importance of mTOR in cell growth, proliferation and survival, research of mTOR in cell adhesion is still in its infancy. Studies have shown that rapamycin inhibits the basal and epidermal growth factor (EGF) stimulated cell adhesion in colon cancer cells (HCT116) [32]. RAD001, a rapamycin analog (rapalog), inhibits collagen or laminin-induced cell adhesion in renal carcinoma cells (A498, Caki-1 and KTC-26) as well [33]. Here we show that rapamycin inhibits the basal and IGF-1 stimulated adhesion of tumor cells derived from human rhabdomyosarcoma (Rh30), Ewing sarcoma (Rh1), colon carcinoma (HT29) and cervical adenocarcinoma (HeLa). Collectively, these findings suggest that mTOR plays a pivotal role in cell adhesion; and mTOR inhibitors may have a potential not only for treatment of cancer, but also for prevention of cancer metastasis. Since the molecular mechanism by which mTOR regulates cell adhesion has not been elucidated, this study was designed to address this question. Here, for the first time, we show that both mTORC1 and mTORC2 are essential for cell adhesion. Interestingly, mTORC1 regulates cell adhesion through S6K1 and 4E-BP1 pathways, but mTORC2 regulates cell adhesion via Akt-independent mechanism.

## RESULTS

### Rapamycin inhibited the basal or IGF-1-stimulated cell adhesion

**Figure 1:**
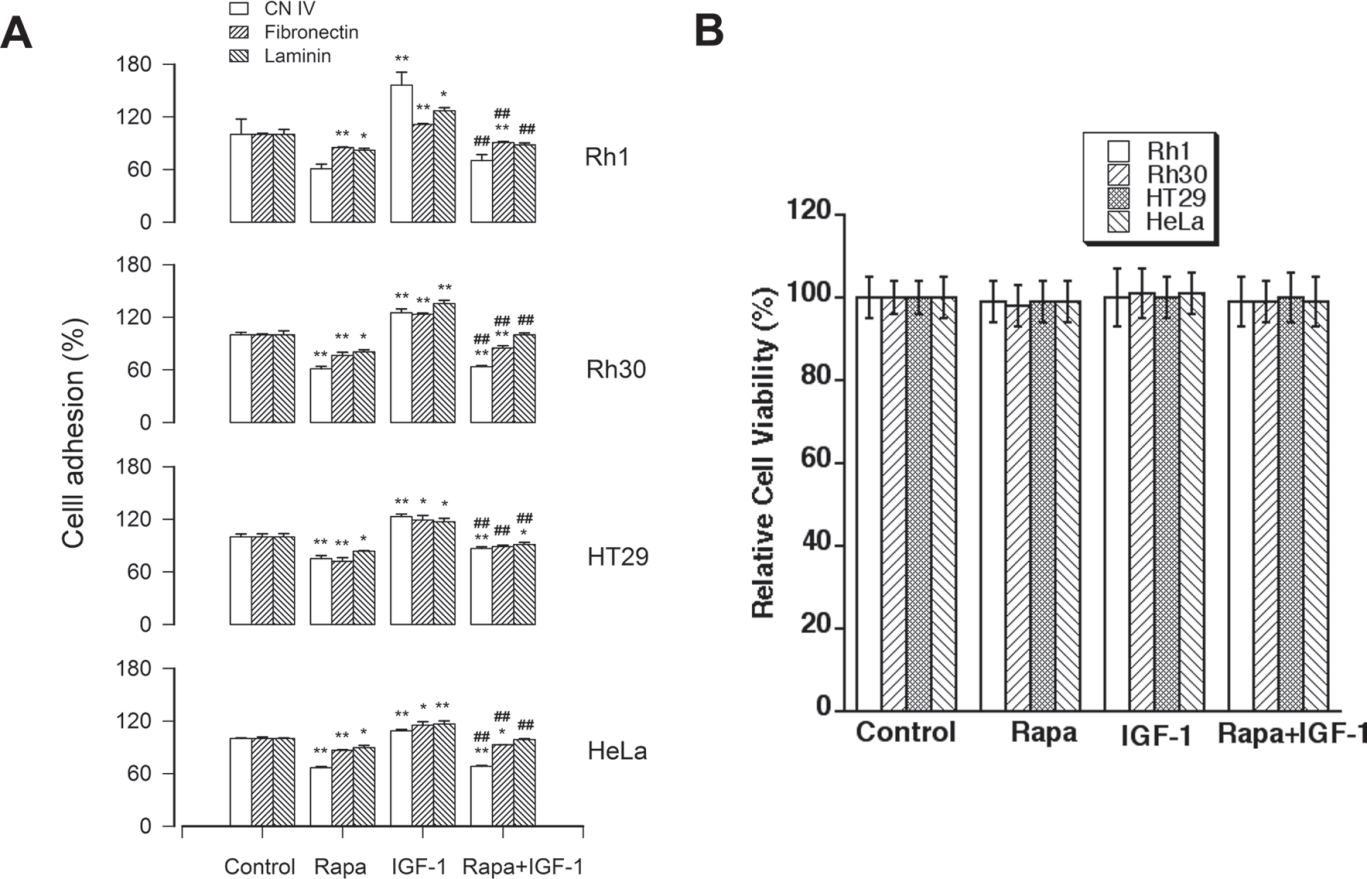
Rapamycin inhibits the basal or IGF-1-stimulated cell adhesion Rh30, Rh1, HT29 and HeLa cells were treated with or without rapamycin (Rapa, 100 ng/ml) in the presence or absence of IGF-1 (10 ng/ml) for 1.5 h following pre-incubation with rapamycin for 2 h, respectively. **(A)** Adherent cells were determined using CN IV-, fibronectin- or laminin-coated cell adhesion assay, and **(B)** cell viability was evaluated by MTS assay, as described in Materials and Methods. Results are means ± SE (*n* = 4–12). *P < 0.05, **P < 0.01, difference *versus* control group. ^##^*P* < 0.01, difference *versus* IGF-1 group.

As mTOR lies downstream of IGF-1 receptor, we focused on assessing the role of mTOR in IGF-1-mediated cell adhesion. For this, human rhabdomyosarcoma (Rh30) and Ewing sarcoma (Rh1) cells were primarily chosen as models, because these cells, under autocrine conditions, are able to produce large quantity of IGF-II, which has approximately equal affinity to IGF-1 receptor as IGF-1 [34–36]. Consistent with the finding in colon cancer cells (HCT116) [32], treatment with rapamycin (100 ng/ml) for 2 h inhibited the basal or IGF-1-stimulated adhesion of Rh1 and Rh30 cells (Figure 1A). Similar results were observed in HT29 and HeLa cells (Figure 1A). The results support the notion that mTOR regulates cell adhesion.

To exclude the possibility that rapamycin inhibits cell adhesion by reducing cell viability, we also examined the effect of rapamycin on cell viability using MTS assay. As shown in Figure 1B, treatment with rapamycin (100 ng/ml) for 4 h did not significantly influence cell viability in all cell lines tested (Rh1, Rh30, HT29 and HeLa). The results indicate that rapamycin inhibits cell adhesion, which is not through reducing cell viability. This is consistent with our previous finding that exposure to rapamycin (100 ng/ml) for ~24 h did not obviously affect cell viability in Rh30 and HeLa cells [20].

### mTOR kinase activity is essential for cell adhesion

**Figure 2:**
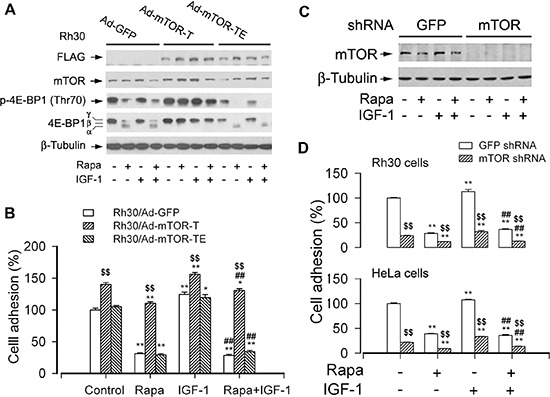
mTOR kinase activity is essential for cell adhesion Serum-starved Rh30 and/or HeLa cells, infected with Ad-mTOR-T, Ad-mTOR-TE, or Ad-GFP (for control), or with lentiviral shRNAs to mTOR or GFP, were treated with or without rapamycin (Rapa, 100 ng/ml) for 2 h, followed by stimulation with or without IGF-1 (10 ng/ml) for 1 h. **(A and C)** Total cell lysates were subjected to Western blotting using indicated antibodies. The blots were probed for β-tubulin as a loading control. Similar results were observed in at least three independent experiments. **(B and D)** Adherent cells were determined using CN IV-coated cell adhesion assay. (A) Western blot analysis showed stable expression of FLAG-tagged mutants of mTOR in Rh30 cells infected with Ad-mTOR-T and Ad-mTOR-TE, but not in the control cells infected with Ad-GFP. Expression of mTOR-T, but not mTOR-TE or GFP, prevented rapamycin inhibition of the basal or IGF-1-stimulated phosphorylation of 4E-BP1 (Thr70) in Rh30 cells. (B) Ectopic expression of mTOR-T strongly increased cell adhesion and conferred high resistance to rapamycin, whereas expression of mTOR-TE remained sensitive to rapamycin. (C) Lentiviral shRNA to mTOR, but not GFP, downregulated mTOR in Rh30 cells. (D) Downregulation of mTOR inhibited the basal and IGF-1-stimulated adhesion in Rh30 and HeLa cells. Results are means ± SE (*n* = 12). *P < 0.05, **P < 0.01, difference *versus* control group. ^##^*P* < 0.01, difference *versus* IGF-1 group. ^$$^*P* < 0.01, Ad-mTOR-T group *versus* Ad-GFP group, or mTOR shRNA group *versus* GFP shRNA group.

Recently we have found that rapamycin inhibited cell motility in an mTOR kinase activity-dependent manner [20, 24, 25]. Cell adhesion is a key step of cell migration [37]. Therefore, we reasoned that rapamycin inhibits cell adhesion by inhibiting the kinase activity of mTOR as well. However, it has been described that mTOR regulates cell differentiation in an mTOR kinase activity-independent manner [38]. To determine whether mTOR regulates cell adhesion requiring its kinase activity, Rh30 cells were infected with recombinant adenoviral vectors encoding GFP (control), FLAG-tagged rapamycin-resistant but kinase active mTOR (S2035T; mTOR-T) or kinase-dead mTOR-T (S2035T/D2357E; mTOR-TE), serum-starved, and treated with or without rapamycin (Rapa, 100 ng/ml) for 2 h, followed by stimulation with or without IGF-1 (10 ng/ml) for 1 h. As expected, expression of mTOR-T, but not mTOR-TE or GFP, prevented rapamycin inhibition of phosphorylation of 4E-BP1 in Rh30 cells, one of the best-characterized downstream effector molecules of mTOR (Figure 2A). The data revealed that mTOR-T functioned as a rapamycin-resistant mutant, and mTOR-TE as a kinase-dead mutant in Rh30 cells, as seen in C2C12 cells [38]. Of interest, ectopic expression of mTOR-T strongly increased cell adhesion and conferred high resistance to rapamycin, whereas expression of a kinase-dead mTOR mutant (mTOR-TE) remained sensitive to rapamycin (Figure 2B), indicating that rapamycin inhibits cell adhesion in an mTOR kinase activity-dependent manner.

To further corroborate the importance of mTOR kinase activity in cell adhesion, mTOR expression was silenced using RNA interference (RNAi) in Rh30 and HeLa cells. As shown in Figure 2C, lentiviral shRNA to mTOR, but not GFP, downregulated mTOR by approximately 90% in Rh30 cells, as detected by Western blotting. Similar results were seen in HeLa cells (data not shown). Importantly, downregulation of mTOR significantly decreased cell adhesion in Rh30 and HeLa cells (Figure 2D). Taken together, our findings strongly suggest that mTOR kinase activity is essential for cell adhesion.

### Disruption of mTORC1 or mTORC2 suppresses cell adhesion

**Figure 3:**
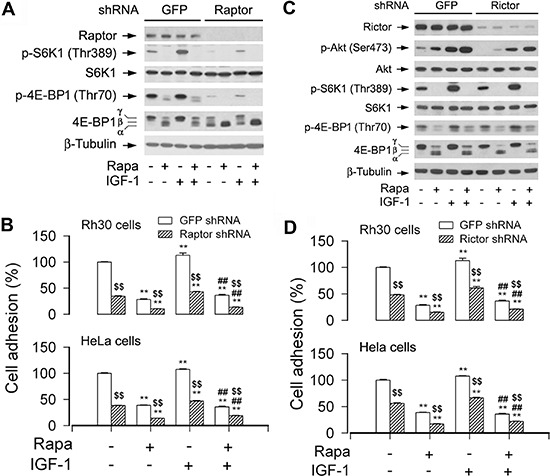
Disruption of mTORC1 or mTORC2 suppresses cell adhesion Serum-starved Rh30 and/or HeLa cells, infected with lentiviral shRNAs to raptor, rictor, or GFP (for control), were treated with or without rapamycin (Rapa, 100 ng/ml) for 2 h, followed by stimulation with or without IGF-1 (10 ng/ml) for 1 h. **(A and C)** Total cell lysates were subjected to Western blotting using indicated antibodies. The blots were probed for β-tubulin as a loading control. Similar results were observed in at least three independent experiments. **(B and D)** Adherent cells were determined using CN IV-coated cell adhesion assay. (A) Lentiviral shRNA to raptor, but not GFP, downregulated raptor and prevented the basal and IGF-1-stimulated phosphorylation of S6K1 and 4E-BP1. (B) Downregulation of raptor inhibited the basal and IGF-1-stimulated cell adhesion in Rh30 and HeLa cells. (C) Lentiviral shRNA to rictor, but not GFP, downregulated rictor and inhibited phosphorylation of Akt (Ser473) in Rh30 cells. (D) Downregulation of rictor inhibited the basal and IGF-1-stimulated cell adhesion in Rh30 and HeLa cells. Results are means ± SE (*n* = 12). **P < 0.01, difference *versus* control group. *^##^*P** < 0.01, difference *versus* IGF-1 group. ^$$^*P* < 0.01, raptor shRNA group or rictor shRNA group *versus* GFP shRNA group.

Two mTOR complexes (mTORC1 and mTORC2) have been identified, but only mTORC1 is sensitive to short rapamycin exposure [3, 4]. In the present study, we have found that treatment with rapamycin for 2 h inhibits cell adhesion (Figure 1). Therefore, we hypothesized that mTORC1 is critical for cell adhesion. To this end, RNAi was utilized to downregulate raptor, an essential component of mTORC1 [3, 4]. As shown in Figure 3A, lentiviral shRNA to raptor, but not GFP, downregulated raptor protein expression by ~90% in Rh30 cells. Consistent with the previous findings [20], downregulation of raptor inhibited the basal and IGF-1-stimulated mTOR-mediated phosphorylation of S6K1 and 4E-BP1 (Figure 3A). Furthermore, downregulation of raptor significantly reduced cell adhesion in Rh30 and HeLa cells (Figure 3B). The data indicate that the integrity of mTORC1 is crucial in the regulation of cell adhesion.

Next, we also tested whether mTORC2 plays a role in the regulation of cell adhesion. Rictor and mSin1 are two essential components of mTORC2, and they interact with and stabilize each other [3, 4]. As expected, rapamycin activated phosphorylation of Akt (S473) in Rh30 cells, but downregulation of rictor by ~90% using lentiviral shRNA to rictor obviously inhibited the basal and IGF-1-stimulated phosphorylation of Akt (S473) (Figure 3C), a substrate of mTORC2 [22]. Interestingly, downregulation of rictor also suppressed cell adhesion in Rh30 and HeLa cells (Figure 3D). The findings suggest that both mTORC1 and mTORC2 control cell adhesion.

**Figure 4:**
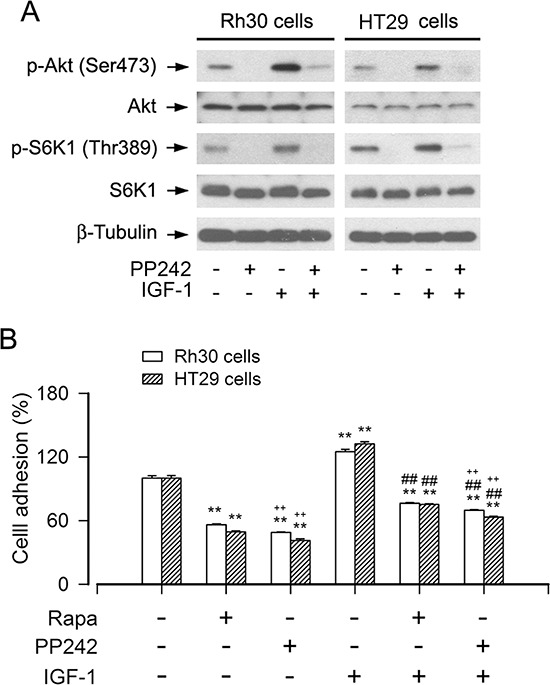
Inhibition of mTORC1/2 by PP242 potently suppresses the basal or IGF-1-stimulated cell adhesion Serum-starved Rh30 and HT29 cells were treated with or without PP242 (1 μM) or rapamycin (Rapa, 100 ng/ml) for 2 h, followed by stimulation with or without IGF-1 (10 ng/ml) for 1 h. **(A)** Total cell lysates were subjected to Western blotting using indicated antibodies, showing that PP242 potently inhibited the basal or IGF-1-stimulated phosphorylation of Akt (Ser473) and S6K1 (Thr389) in Rh30 and HT29 cells. The blots were probed for β-tubulin as a loading control. Similar results were observed in at least three independent experiments. **(B)** Cell adhesion was determined using CN IV-coated cell adhesion assay, showing that inhibition of mTORC1/2 by PP242 dramatically suppressed the basal and IGF-1-stimulated adhesion in Rh30 and HT29 cells, and the inhibitory effect of PP242 was more potent that of Rapa. Results are means ± SE (*n* = 6). **P < 0.01, difference *versus* control group; ^##^*P* < 0.01, difference *versus* IGF-1 group; ^++^*P* < 0.01, PP242 group or PP242+IGF-1 group *versus* Rapa group or Rapa+IGF-1 group.

Since rictor also has mTORC2-independent functions related to regulation of cytoskeleton and cell migration [39–42], to confirm the role of mTORC2 in the regulation of cell adhesion, PP242, an mTOR kinase inhibitor that blocks both mTORC1 and mTORC2, was used. As predicted, PP242 inhibited both mTORC1-mediated phosphorylation of S6K1 and mTORC2-mediated phosphorylation of Akt in Rh30 and HT29 cells (Figure 4A). Interestingly, PP242 (inhibition of mTORC1/2) suppressed the basal or IGF-1-stimulated cell adhesion of Rh30 and HT29 cells more potently than rapamycin (inhibition of mTORC1) (Figure 4B). Collectively, the above results demonstrate that both mTORC1 and mTORC2 are involved in the regulation of cell adhesion.

### S6K1 pathway is essential for mTOR-mediated cell adhesion

**Figure 5:**
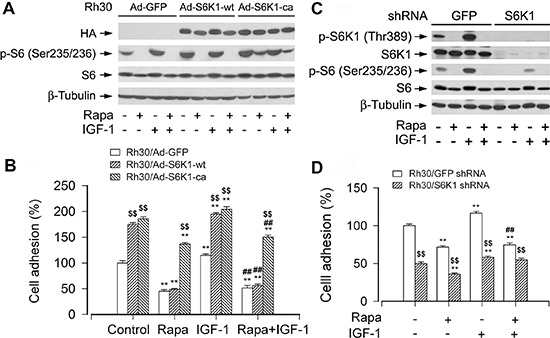
mTORC1-mediated S6K1 pathway is involved in the regulation of cell adhesion Serum-starved Rh30 cells, infected with Ad-S6K1-wt, Ad-S6K1-ca, or Ad-GFP (for control), were treated with or without rapamycin (Rapa, 100 ng/ml) for 2 h, followed by stimulation with IGF-1 (10 ng/ml) for 1 h. **(A and C)** Total cell lysates were subjected to Western blotting using indicated antibodies. The blots were probed for β-tubulin as a loading control. Similar results were observed in at least three independent experiments. **(B and D)** Adherent cells were determined using CN IV-coated cell adhesion assay. (A) and (B) Rh30 cells expressing S6K1-ca, but not S6K1-wt or GFP, were resistant to rapamycin inhibition of phosphorylation of S6, as well as basal and IGF-1-stimulated cell adhesion. (C) Lentiviral shRNA to S6K1, but not GFP, downregulated S6K1 in Rh30 cells. (D) Downregulation of S6K1 significantly inhibited the basal and IGF-1-stimulated cell adhesion in Rh30 cells. Results are means ± SE (*n* = 12). **P < 0.01, difference *versus* control group; ^##^*P* < 0.01, difference *versus* IGF-1 group; ^$$^*P* < 0.01, Ad-S6K1-wt or Ad-S6K1-ca group *versus* Ad-GFP group, or S6K1 shRNA group *versus* GFP shRNA group.

To understand how mTORC1 mediates cell adhesion, next, we investigated the effect of S6K1, one of the best-characterized downstream effector molecules of mTORC1, on cell adhesion [3, 4]. To this end, Rh30 cells were firstly infected with recombinant adenoviruses expressing HA-tagged wild-type (wt) S6K1 (Ad-S6K1-wt), constitutively active and rapamycin-resistant S6K1 mutant of S6K1 (Ad-S6K1-ca) and control virus encoding GFP alone. As shown in Figure 5A, high levels of recombinant S6K1-wt or S6K1-ca in the cells infected with Ad-S6K1-wt or Ad-S6K1-ca, but not in the cells infected with Ad-GFP, were seen as determined by Western blotting. Of note, cells expressing S6K1-wt or S6K1-ca, but not GFP, had a robust phosphorylation of S6 ribosomal protein (p-S6), a substrate of S6K1. Addition of IGF-1 failed to further enhance the phosphorylation of S6. Possibly, the constitutively active S6K1 saturated the phosphorylation of S6, or alternatively, the constitutively active S6K1 blocked the action of IGF-1 through the S6K-IRS feedback loop [3]. Nevertheless, cells expressing S6K1-ca, but not S6K1-wt or GFP, were resistant to rapamycin inhibition of phosphorylation of S6. Similarly, cells expressing S6K1-ca, but not S6K1-wt or GFP, were resistant to rapamycin inhibition of the basal and IGF-1-stimulated cell adhesion (Figure 5B), suggesting that mTOR positively regulates cell adhesion, at least, requiring a certain level of S6K1 activity.

In addition, we also investigated whether expression of constitutively active S6K1 is able to attenuate the inhibitory effect of PP242 on cell adhesion. As predicted, treatment with rapamycin (100 ng/ml) for 2 h only inhibited mTORC1 in the control cells infected with Ad-GFP, whereas treatment with PP242 (1 μM) for 2 h inhibited both mTORC1 and mTORC2 very potently, since both mTORC1-mediated p-S6K1 (Thr389) and mTORC2-mediated p-Akt (Ser473) were almost completely blocked in the control cells infected with Ad-GFP (Figure S1A). In consistence with the findings in Figure 5A and 5B, expression of constitutively active and rapamycin-resistant S6K1 (S6K1-ca), but not GFP (control), conferred resistance to rapamycin inhibition of p-S6 and cell adhesion (Figure S1A and S1B). Of interest, expression of S6K1-ca also rendered resistance to PP242 inhibition of p-S6 and cell adhesion, although the resistance to PP242 was weaker than that to rapamycin (Figure S1). The results further support that S6K1 plays a critical role in mTOR-mediated cell adhesion.

To substantiate the role of S6K1 in cell adhesion, lentiviral shRNA to S6K1 was used to silence expression of S6K1 in Rh30 cells. As demonstrated in Figure 5C, infection of Rh30 cells with lentiviral shRNA to S6K1 for 5 days downregulated expression of cellular S6K1 protein by ~80% compared to control cells (infected with lentiviral shRNA to GFP). In line with this, silencing S6K1 expression resulted in reduction of S6K1 kinase activity, as detected by Western blotting with antibodies to phospho-S6K1 (T389) and phospho-S6 ribosomal protein (S235/236), respectively (Figure 5C). Consequently, the adhesion of Rh30 cells treated with S6K1 shRNA was significantly inhibited (Figure 5D). The results further indicate that S6K1 pathway is essential for mTOR-mediated cell adhesion.

### 4E-BP1/eIF4E pathway is necessary for mTOR-mediated cell adhesion

**Figure 6:**
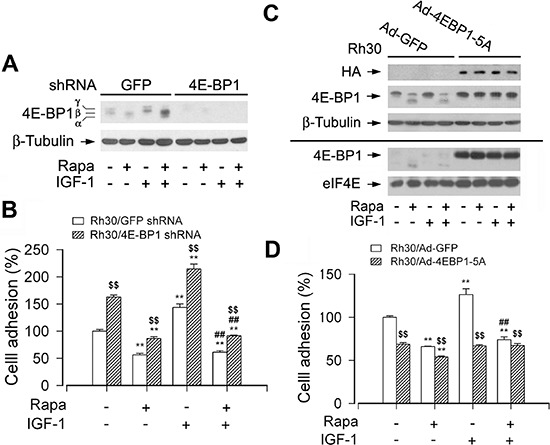
mTORC1-mediated 4E-BP1 pathway is involved in the regulation of cell adhesion Serum starved Rh30 cells, infected with lentiviral shRNAs to 4E-BP1 or GFP (for control), or with Ad-4EBP1-5A and Ad-GFP, were treated with or without rapamycin (Rapa, 100 ng/ml) for 2 h, followed by stimulation with or without IGF-1 (10 ng/ml) for 1 h. **(A and C)** Total cell lysates were subjected to Western blotting using indicated antibodies. The blots were probed for β-tubulin as a loading control. Similar results were observed in at least three independent experiments. **(B and D)** Adherent cells were determined using CN IV-coated cell adhesion assay. (A) Lentiviral shRNA to 4E-BP1, but not GFP, resulted in a significant downregulation of 4E-BP1 in Rh30 cells. (B) Downregulation of 4E-BP1 significantly elevated the basal and IGF-1-stimulated cell adhesion in Rh30 cells. (C) Exposure to rapamycin due to HA-tagged 4E-BP1 in Ad-4EBP1-5A-infected cells failed to change the mobility of 4E-BP1-5A (upper panel). However, using 7-methyl GTP-Sepharose pull-down assay, very striking amount of recombinant 4EBP1-5A was observed to bind to eIF4E, even in the absence of rapamycin (bottom panel). (D) Expression of 4EBP1-5A inhibited the basal and IGF-1-stimulated cell adhesion in Rh30 cells. Results are means ± SE (*n* = 12). **P < 0.01, difference *versus* control group; ^##^*P* < 0.01, difference *versus* IGF-1 group; ^$$^*P* < 0.01, GFP shRNA group *versus* 4E-BP1 shRNA group, or Ad-4EBP1-5A group *versus* Ad-GFP group.

4E-BP1/eIF4E is another best-known downstream signaling pathway of mTORC1 [3, 4]. Next, we further evaluated the role of 4E-BP1/eIF4E pathway in the regulation of cell adhesion. As shown in Figure 6A, infection of Rh30 cells with lentiviral shRNA to 4E-BP1 resulted in a significant downregulation of 4E-BP1 protein expression compared to control cells infected with lentiviral shRNA to GFP, as detected by Western blotting with antibodies to 4E-BP1. Consequently, the adhesion of 4E-BP1-downregulaed Rh30 cells was significantly elevated (Figure 6B), suggesting that mTOR regulates cell adhesion also at least in part by 4E-BP1/eIF4E pathway.

To further confirm the functional significance of 4E-BP1/eIF4E pathway in the mTORC1-mediated cell adhesion, next, we employed recombinant adenovirus encoding HA-tagged 4E-BP1 mutants where Thr36, Thr45, Ser64, Thr69 and Ser82 are replaced by Ala residues (designated 4EBP1-5A) mimicking hypophosphorylated residues, thus tightly binding to and sequester eIF4E in cells [31]. As shown in Figure 6C, HA-tagged 4E-BP1 and higher levels of 4E-BP1 were detected in Ad-4EBP1-5A-infected cells. Exposure to rapamycin failed to change the mobility of 4E-BP1-5A (Figure 6C, upper panel). However, using 7-methyl GTP-Sepharose pull-down assay, very striking amount of recombinant 4EBP1-5A was observed to bind to eIF4E, even in the absence of rapamycin (Figure 6C, bottom panel). The results clearly demonstrate that Ad-4EBP1-5A functions as a dominant suppressor of eIF4E in the cells. Interestingly, expression of 4EBP1-5A inhibited the basal and IGF-stimulated cell adhesion (Figure 6D). The finding verifies that 4E-BP1/eIF4E pathway is as critical as S6K1 pathway for mTOR-mediated cell adhesion.

### Akt does not contribute to mTOR-mediated cell adhesion

**Figure 7:**
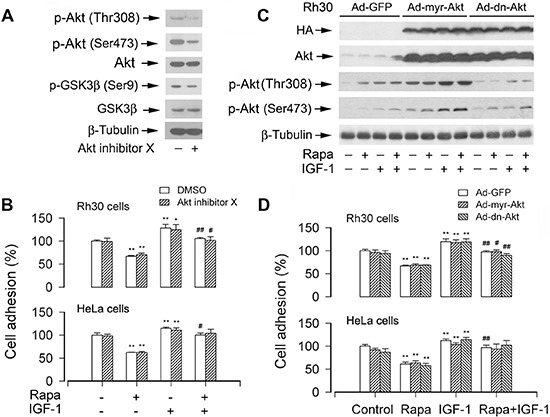
Pharmacological inhibition of Akt or ectopic expression of constitutively active or dominant negative Akt does not affect cell adhesion **(A)** Serum-starved Rh30 were treated with or without Akt inhibitor X (10 μM) for 2 h, followed by Western blot analysis using indicated antibodies, showing that Akt inhibitor X inhibited the phosphorylation of Akt and its substrate GSK3β in the cells. **(B)** The adhesion of Rh30 and HeLa cells, treated with or without rapamycin and/or IGF-1 following pre-incubation with or without Akt inhibitor X for 2 h, was not significantly affected. **(C and D)** Serum starved Rh30 and/or HeLa cells, infected with Ad-myr-Akt, Ad-dn-Akt, or Ad-GFP (for control), were treated with or without rapamycin (Rapa, 100 ng/ml) for 2 h, followed by stimulation with or without IGF-1 (10 ng/ml) for 1 h. (C) Total cell lysates were subjected to Western blotting using indicated antibodies. The blots were probed for β-tubulin as a loading control. Similar results were observed in at least three independent experiments. (D) Adherent cells were determined using CN IV-coated cell adhesion assay, showing that ectopic expression of myr-Akt or dn-Akt did not exhibit an obvious stimulatory or inhibitory effect on cell adhesion in Rh30 and HeLa cells. Results are means ± SE (*n* = 12). *P < 0.05, **P < 0.01, difference *versus* control group; ^#^*P* < 0.01, ^##^*P* < 0.01, difference *versus* IGF-1 group.

We have displayed that mTORC2 controls cell adhesion (Figure 3). It has been reported that mTORC2 directly phosphorylates Akt on Ser473 [22]. Akt is a well characterized substrate of mTORC2 [3, 4]. Therefore, we asked whether mTORC2-mediated Akt pathway plays an important role in the regulation of cell adhesion. For this, Akt inhibitor X, a selective Akt inhibitor, was utilized. We found that treatment with Akt inhibitor X (10 μM) for 2 h inhibited phospho-Akt and phospho-GSK3β in the cells, as detected by Western blot analysis (Figure 7A). Of note, the adhesion of Rh30 cells, treated with or without rapamycin and/or IGF-1 after pre-incubation with or without Akt inhibitor X for 2 h, was not significantly affected (Figure 7B). Similar data were also seen in HeLa cells (Figure 7B). The results imply that Akt may not contribute to mTORC2-mediated cell adhesion.

To corroborate the above finding, recombinant adenoviruses encoding HA-tagged constitutively active Akt (Ad-myr-Akt) and dominant negative Akt (Ad-dn-Akt), respectively, were employed. Infection of Rh30 cells with Ad-myr-Akt and Ad-dn-Akt, but not Ad-GFP (control virus), resulted in expression of high levels of HA-tagged Akt mutants (Figure 7C). Expression of myr-Akt, but not expression of dn-Akt, led to robust phosphorylation of Akt (Ser473) even without stimulation with rapamycin and/or IGF-1 (Figure 7C), indicating that the Akt mutants were functional in the cells as expected. Surprisingly, ectopic expression of myr-Akt or dn-Akt did not obviously stimulate or inhibit cell adhesion in Rh30 and HeLa cells treated with or without rapamycin and/or IGF-1 (Figure 7D). The results clearly indicate that mTORC2 regulates cell adhesion independently of Akt.

## DISCUSSION

Migration of cancer cells is under intense investigation as a prerequisite for cancer metastasis, which is a primary cause of mortality in most *cancer* patients [1, 2, 43–47]. As cell migration is a multistep cellular event, including cell polarization/protrusion, adhesion and de-adhesion [37], disturbance of any of these steps may intervene cancer metastasis. Therefore, it is of great importance to find a novel therapeutic target and strategy to control cancer metastasis in individuals with cancer. We have shown that rapamycin inhibits cell motility by suppression of mTOR-mediated S6K1 and 4E-BP1 pathways [20], and identified that rapamycin inhibition of cell motility is related to its prevention of F-actin reorganization [24, 25]. Here, for the first time, we show that both mTORC1 and mTORC2 are essential for cell adhesion. Furthermore, mTORC1 regulates cell adhesion via S6K1 and 4E-BP1 pathways, but mTORC2 regulates cell adhesion through Akt-independent mechanism.

It is well known that mTOR functions as a central controller of cell growth, proliferation, differentiation and survival [3, 4]. Increasing evidence implicates that mTOR pathway also plays a crucial role in the regulation of tumor cell motility and invasion, as well as cancer metastasis [20, 24, 25, 48, 49]. Rapamycin suppresses tumor cell growth/proliferation [3, 4] and motility [20, 24, 25], and induces apoptosis of tumor cells under certain conditions [50, 51] by inhibiting the kinase activity of mTOR. Previous studies have also shown that rapamycin inhibits the basal and epidermal growth factor (EGF) stimulated cell adhesion in colon cancer cells (HCT116) [32]. RAD001, a rapalog, has also been found to inhibit collagen or laminin-induced cell adhesion in renal carcinoma cells (A498, Caki-1 and KTC-26) [33]. In the present study, we further observed that allosteric inhibition of mTORC1 by rapamycin suppressed IGF-1-stimulated cell adhesion in a panel of tumor cell lines, including human rhabdomyosarcoma (Rh30), Ewing sarcoma (Rh1), colon carcinoma (HT29), and cervical adenocarcinoma (HeLa) cells, which was not by reducing the cell viability. In addition, inhibition of mTORC1/2 kinase activity by PP242 exhibited more potent inhibitory effect on cell adhesion in the tumor cells. The findings from this group and others [32, 33] strongly support the concept that mTOR regulates cell adhesion, which is independent of cancer cell lines or stimuli.

Cell adhesion is a key step during cell migration [37]. Since it has been shown that mTOR regulates cell differentiation independently of mTOR kinase activity [38, 52], although there exist disputations [53, 54], this prompted us to study whether rapamycin inhibits cell adhesion in an mTOR kinase activity-dependent manner. We found that expression of a rapamycin-resistant but kinase active mTOR (S2035T; mTOR-T), but not kinase-dead mTOR-T (S2035T/D2357E; mTOR-TE), prevented rapamycin from inhibiting IGF-1-stimulated cell adhesion (Figure 2B), revealing that mTOR kinase activity is essential for cell adhesion. This is further supported by the observations that the adhesion of Rh30 and HeLa cells treated with mTOR shRNA or PP242 (an mTOR kinase inhibitor) was profoundly inhibited. Taken together, our data underscore a critical role of mTOR in cell adhesion.

Studies have identified two structurally and functionally distinct mTOR-containing multiprotein complexes (mTORC1 and mTORC2) [3, 4]. The functions of mTORC1 and mTORC2 are greatly affected by the complex integrity, especially their associations with raptor [6, 7] and rictor [11, 12], respectively. mTORC1 regulates phosphorylation of S6K1 and 4E-BP1 [6, 7], and mTORC2 phosphorylates Akt at S473 [22]. Most functions of mTORC1 are sensitive to rapamycin, and mTORC1 controls translation initiation, ribosome biogenesis and other growth-related events [3, 4]. However, the action of rapamycin on mTORC2-mediated Akt depends on the concentration and duration of rapamycin treatment, and mTORC2 regulates polarization of actin cytoskeleton [11, 12]. Here we found that disruption of mTORC1 or mTORC2 by silencing raptor or rictor, respectively, inhibited the basal and IGF-1-stimulated adhesion of cancer cells. Furthermore, PP242 (an mTORC1/2 kinase inhibitor) inhibited cell adhesion more potently than rapamycin (an mTORC1 inhibitor), suggesting that both mTORC1 and mTORC2 regulate cell adhesion.

S6K1 and 4E-BP1 are two best-characterized downstream targets of mTORC1 [3, 4]. To gain more insights into in the event that mTORC1 regulates cell adhesion, we dissected the roles of these two downstream pathways in the regulation of cell adhesion. The levels or activities of S6K1 and 4E-BP1 were individually manipulated genetically. Cells infected with an adenoviral recombinant expressing constitutively active and rapamycin-resistant mutant of S6K1 (Ad-S6K1-ca), but not with an adenovirus expressing wild-type S6K1 (Ad-S6K1-wt), or a control viral vector (Ad-GFP), conferred to resistance to rapamycin, and cell adhesion was rescued. Consistently, IGF-1 failed to stimulate adhesion in the S6K1-downregulated cells. On the other hand, downregulation of 4E-BP1 by shRNA greatly attenuated the inhibitory effect of rapamycin on cell adhesion. In contrast, genetic expression of constitutively hypophophorylated 4E-BP1 (4E-BP1-5A) potently inhibited IGF-1-stimulated cell adhesion. The findings highlight that both mTORC1-mediated S6K1 and 4E-BP1 pathways are essential for cell adhesion.

A new question that arises from this work is how mTORC1-mediated S6K1 and 4E-BP1 pathways regulate cell adhesion. It is well known that both S6K1 and 4E-BP1/eIF4E pathways contribute to protein synthesis [3, 4]. Cell adhesion is mediated by multiple signaling molecules, such as integrins, E-cadherin, focal adhesion kinase (FAK), Src, integrin-linked kinase, the small GTPases (RhoA, Rac1, Cdc42), protein phosphatase 2A, and Erk1/2 [32, 55–58]. Whether mTORC1-mediated S6K1 and 4E-BP1 pathways regulate cell adhesion by impacting those proteins at transcriptional, translational or post-translational level remains to be defined. Obviously, more studies are required to address the questions.

In the studies, we found that silencing rictor inhibited cell adhesion, suggesting that mTORC2 may participate in the regulation of cell adhesion. Since rictor is not only essential for mTORC2 function, but also carries mTORC2-independent functions related to regulation of cytoskeleton and cell migration [39–42], in order to verify our finding that the inhibition of cell adhesion by rictor shRNA indeed represents the role of mTORC2 in the regulation of cell adhesion, we took advantage of the mTORC1/2 inhibitor PP242. Of interest, PP242 suppressed the basal or IGF-1-stimulated cell adhesion of Rh30 and HT29 cells more potently than rapamycin (mTORC1 inhibitor) (Figure 4). The data support the conclusion that both mTORC1 and mTORC2 are involved in the regulation of cell adhesion.

As Akt is a well characterized substrate of mTORC2 [26], we therefore speculated that the Akt pathway is involved in regulating cell adhesion. However, to our surprise, the experiments using genetic manipulation or pharmacological inhibition of Akt activity demonstrated that mTOR-mediated cell adhesion was independent of Akt pathway. This is in contrast to the observation that Akt activity is important for stromal cell-derived factor-1 (SDF-1)-induced hematopoietic progenitor cell adhesion to bone marrow stromal cells [59]. The discrepancy is likely due to the different cell lines or experimental conditions used. Noticeably, although Akt positively regulates motility in many cell lines [60, 61], it negatively regulates SDF-1-mediated migration of hematopoietic progenitors [59]. Since mTORC2 not only regulates phosphorylation of Akt, but also regulates phosphorylation of serum- and glucocorticoid-induced protein kinase 1 (SGK1) [19], focal adhesion proteins (FAK, paxillin, and p130^Cas^) [18, 25] and protein kinase C-α [17], the activities of small GTPases (RhoA, Cdc42 and Rac1) [18, 24, 58], further research is required to determine whether mTORC2 regulates cell adhesion by mediating any of these signaling molecules.

**Figure 8:**
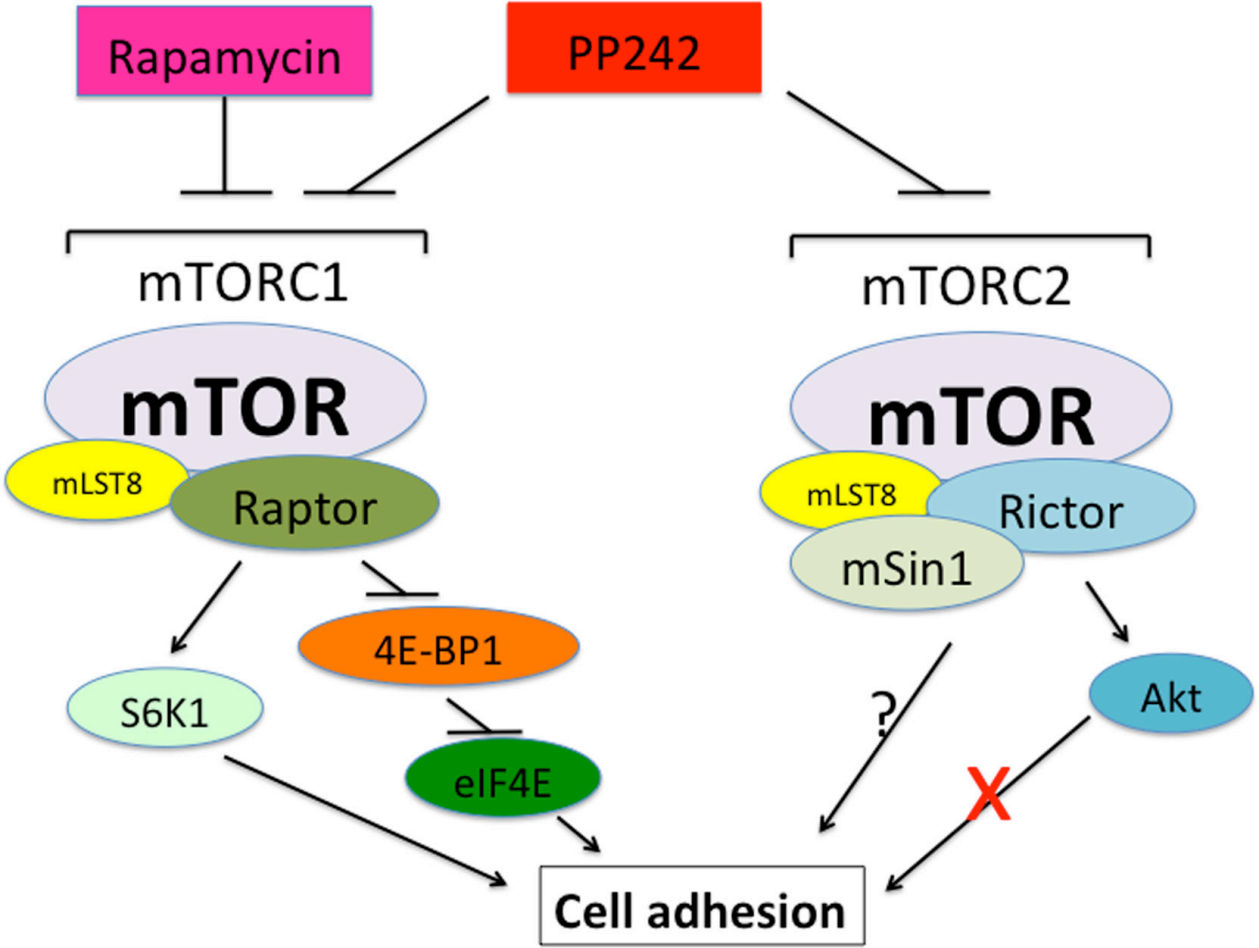
A schematic model showing how mTOR regulates cell adhesion Both mTORC1 and mTORC2 control cell adhesion. mTORC1 regulates cell adhesion through S6K1 and 4E-BP1/eIF4E pathways, but mTORC2 mediates cell adhesion independently of Akt.

In summary, we have demonstrated that rapamycin inhibits the basal or IGF-1-stimulated adhesion of cancer cells. mTOR kinase activity is essential for cell adhesion. Both mTORC1 and mTORC2 participate in the regulation of cell adhesion (Figure 8). However, mTORC1 regulates cell adhesion through S6K1 and 4E-BP1 pathways, but mTORC2 regulates cell adhesion by Akt-independent mechanism. Further research is needed to address how mTORC2 regulates cell adhesion.

## MATERIALS AND METHODS

### Reagents

Rapamycin (LC Laboratories, Woburn, MA, USA), PP242 and Akt inhibitor X (Santa Cruz Biotechnology, Santa Cruz, CA, USA) were dissolved in dimethyl sulfoxide (DMSO) to prepare 100 μg/ml rapamycin, 1 mM PP242 and 10 mM Akt inhibitor X as stock solutions, and stored at −20°C. IGF-1 (PeproTech, Rocky Hill, NJ, USA) was rehydrated in 0.1 M acetic acid to prepare a 10 μg/ml stock solution and stored at −80°C. Bovine collagen type IV (CN IV) was purchased from Rockland Immunochemicals (Gilbertsville, PA, USA). 7-methyl GTP Sepharose 4B was from GE Healthcare Life Sciences (Piscataway, NJ, USA). CellTiter 96^®^ AQ_ueous_ One Solution Cell Proliferation Assay Kit was from Promega (Madison, WI, USA). Enhanced chemiluminescence solution was from Pierce (Rockford, IL, USA). Antibodies included those against S6K1, Akt, S6, eIF4E, GSK3β, HA (Santa Cruz Biotechnology, Santa Cruz, CA, USA), mTOR, phospho-S6K1 (Thr389), phospho-Akt (Ser473), phospho-Akt (Thr308), phospho-S6 (Ser/235/236), 4E-BP1, phospho-4E-BP1 (Thr70) (Cell Signaling, Beverly, MA, USA), raptor, rictor (Bethyl Laboratories, Montgomery, TX, USA), phospho-GSK3β (Ser9) (Epitomics, Burlingame, CA, USA), β-tubulin, FLAG (Sigma, St. Louis, MO, USA), goat anti-rabbit IgG-horseradish peroxidase (HRP), goat anti-mouse IgG-HRP, and rabbit anti-goat IgG-HRP (Pierce). All other chemicals were purchased from Sigma.

### Cell lines and cultures

Cell lines from human rhabdomyosarcoma (Rh30) and Ewing sarcoma (Rh1) (obtained from Dr. Peter J. Houghton, St. Jude Children's Research Hospital, Memphis, TN, USA, in 2003) were grown in antibiotic-free RPMI 1640 medium (Mediatech, Herndon, VA, USA) supplemented with 10% fetal bovine serum (FBS) (Hyclone, Logan, UT, USA). Colon carcinoma (HT29) and cervical adenocarcinoma (HeLa) cells (obtained from American Type Culture Collection, Manassas, VA, USA, in 2004) were grown in antibiotic-free Dulbecco's modified Eagle medium (DMEM) (Mediatech, Herndon, VA, USA) supplemented with 10% FBS. All cells were maintained in a humid incubator (37°C, 5% CO_2_). For experiments where cells were deprived of serum, cell monolayers were washed with phosphate-buffered saline (PBS), and incubated in the serum-free DMEM (Mediatech).

### Recombinant adenoviral constructs and infection of cells

Recombinant adenoviral vectors encoding green fluorescence protein (Ad-GFP), FLAG-tagged rapamycin resistant mTOR mutant (S2035T; mTOR-T), kinase dead mTORrr mutant (S2035T/D2357E; mTOR-TE), hemagglutinin (HA)-tagged constitutively hypophosphorylated 4E-BP1 (Ad-4EBP1-5A), wild-type S6K1 (Ad-S6K1-wt) and constitutively active and rapamycin-resistant S6K1 (Ad-S6K1-ca) were described previously [20, 24, 25]. Recombinant adenoviral vectors encoding HA-tagged dominant negative Akt (dn-Akt, T308A/S473A) and constitutively active Akt (myr-Akt) [62] (generously provided from Kenneth Walsh, Boston University, Boston, MA). For experiments, Rh1 and Rh30 cells were grown in six-well plates in RPMI 1640 medium supplemented with 10% FBS, and infected with the individual adenovirus for 24 h at 1 of multiplicity of infection (MOI = 1). Subsequently, cells were washed with PBS and serum starved in DMEM for 24 h before experiments. Ad-GFP alone served as a control. Expression of HA-tagged 4EBP1-5A, S6K1-wt, S6K1-ca, dn-Akt or myr-Akt, and FLAG-tagged mTOR-T or mTOR-TE was determined by Western blot with antibodies to HA and FLAG, respectively. The function of 4E-BP1-5A was examined by analysis of 4E-BP1-eIF4E binding using 7-methyl GTP Sepharose pull-down assay [20], whereas the function of S6K1-wt, S6K1-ca, mTOR-T and mTOR-TE was determined by immunoblotting with antibodies to S6K1, phospho-S6 ribosomal protein (Ser235/236), and phospho-4E-BP1 (Thr70), respectively.

### Lentiviral shRNA cloning, production and infection

Lentiviral shRNAs to GFP, mTOR, raptor, rictor, S6K1 and 4E-BP1 were described previously [20, 24, 25]. The lentivirus-expressing GFP-target shRNA was used as control. Monolayer Rh30 or HeLa cells, when grown to about 70% confluence, were infected with above lentivirus-containing supernatant in the presence of 8 μg/ml polybrene and, exposed to 2 μg/ml puromycin after 24 h of infection. In 5 days, cells were used for experiments.

### Western blot analysis

Western blotting was performed, as described previously [63].

### Cell viability assay

Cell viability was evaluated using MTS assay, as described previously [64]. Briefly, cells suspended in the growth medium were seeded in a 96-well plate at a density of 1 × 10^4^ cells/well (in 6 replicates) and were grown overnight at 37°C in a humidified incubator with 5% CO_2_. After serum-starvation for 24 h, the cells were treated with or without rapamycin (100 ng/ml) for 4 h, followed by MTS assay.

### Cell adhesion assay

For collagen type IV (CN IV)-coated assay, cell adhesion was evaluated as described previously [32], with some modifications. Briefly, 96-well tissue culture plates were coated for 2 h at 37°C with CN IV at a concentration of 0.2 μg/ml, followed by blocking with 3% bovine serum albumin for 3 h, and then rinsed once with PBS. Cells for determination of adhesion functions were changed to serum-free DMEM and grown for 24 h. After trypsinization, serum-starved cells were incubated at a density of 1.2 × 10^6^ cells/ml at 37°C with/without 100 ng/ml rapamycin for 2 h. The cells were then plated at 6 × 10^4^ cells/well on CN IV-coated plates and incubated for 1.5 h in the absence or presence of rapamycin (100 ng/ml) and/or IGF-1 (10 ng/ml). Non-adherent cells were removed by washing three times with serum-free DMEM. Afterwards, each well was added 20 μl of one solution reagent (Promega) and incubated for 4 h. The relative number of attached cells was determined by measuring the optical density (OD) at 490 nm using a Wallac 1420 Multilabel Counter (PerkinElmer Life Sciences, Wellesley, MA, USA). For fibronectin- or laminin-coated cell adhesion assay, adherent cells were detected using the CytoSelect™ Cell Adhesion Assay kit (Cell Biolabs, San Diego, CA, USA) according to the protocols supplied by the manufacturer.

### Statistical analysis

Results were expressed as mean values ± standard error (Means ± SE). The data were analyzed by one-way analysis of variance (ANOVA) followed by post-hoc Dunnett's *t*-test for multiple comparisons. A level of *P* < 0.05 was considered to be statistically significant.

## SUPPLEMENTARY FIGURE


