# Supplementary material for: Both mTORC1 and mTORC2 are involved in the regulation of cell adhesion
Source: Oncotarget. 2015 Jan 23;6(9):7136–50. doi: 10.18632/oncotarget.3044 (PMC4466674; doi:10.18632/oncotarget.3044)
Supplement: Supplementary file 1 [file oncotarget-06-7136-s001.pdf]

SUPPLEMENTARY FIGURE

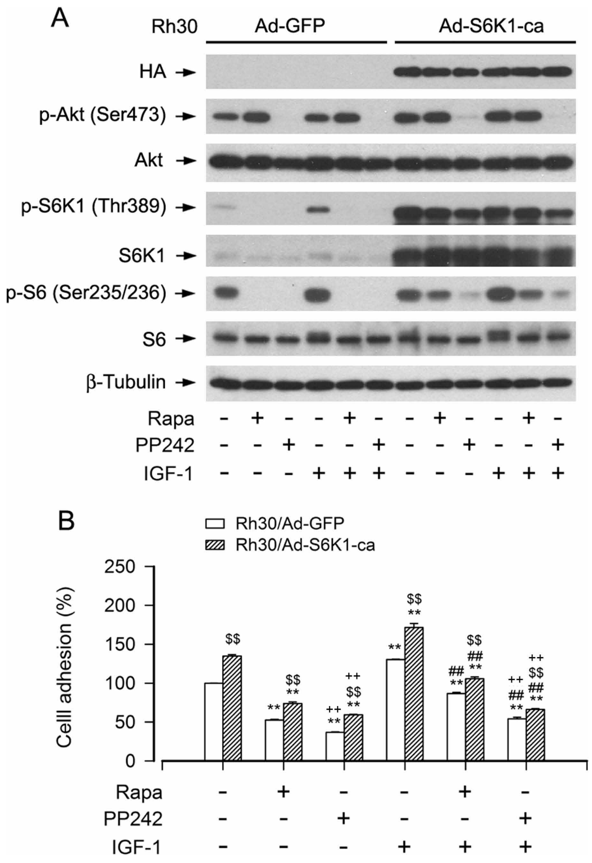

Supplementary Figure S1: Expression of constitutively active and rapamycin-resistant S6K1 prevents PP242 or rapamycin from inhibiting cell adhesion. Serum-starved Rh30 cells, infected with Ad-S6K1-ca, or Ad-GFP (for control), were treated with or without PP242 (1 μM) or rapamycin (Rapa, 100 ng/ml) for 2 h, followed by stimulation with or without IGF-1 (10 ng/ml) for 1 h. **(A)** Total cell lysates were subjected to Western blotting using indicated antibodies. The blots were probed for β-tubulin as a loading control. Similar results were observed in at least three independent experiments. **(B)** Cell adhesion was determined using CN IV-coated cell adhesion assay. (A and B) Rh30 cells expressing S6K1-ca, but not GFP, were resistant to PP242 or rapamycin inhibition of phosphorylation of S6K1 and S6, as well as basal and IGF-1-stimulated cell adhesion. Results are means ± SE (n = 6). $^{**}P < 0.01$, difference *versus* control group; $^{\#\#}P < 0.01$, difference *versus* IGF-1 group; $^{\$\$}P < 0.01$, Ad-S6K1-ca group *versus* Ad-GFP group; $^{++}P < 0.01$, PP242 group or PP242+IGF-1 group *versus* Rapa group or Rapa+IGF-1 group.
